# Supplementary material for: Foliar Selenium Application to Reduce the Induced-Drought Stress Effects in Coffee Seedlings: Induced Priming or Alleviation Effect?
Source: Plants (Basel). 2023 Aug 23;12(17):3026. doi: 10.3390/plants12173026 (PMC10490047; doi:10.3390/plants12173026)

Supplementary material

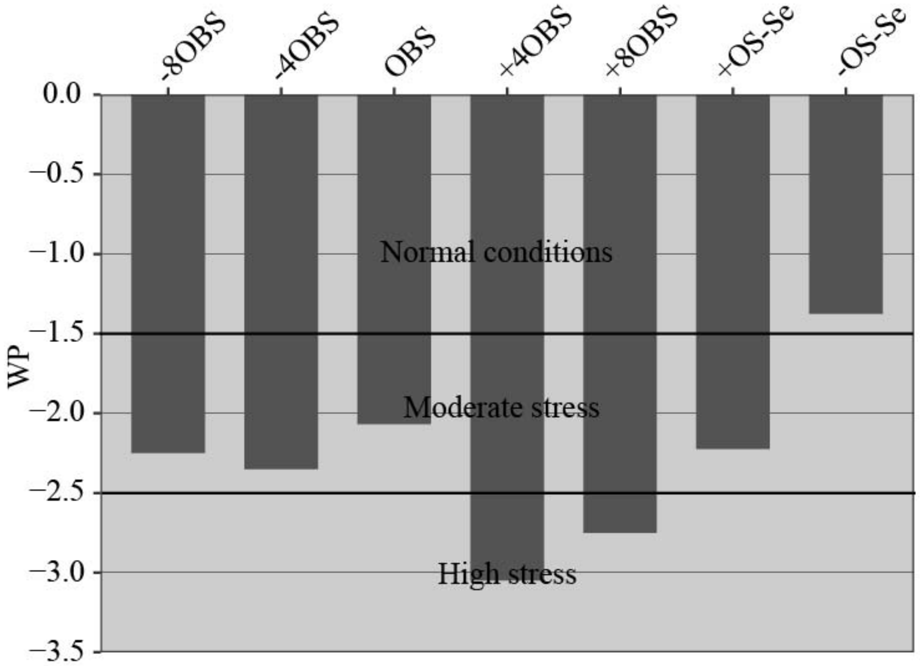

Supplementary Figure S1: Water potential of coffee leaves as a result of Se application in C. arabica cv. Catuai seedlings under osmotic stress induced by PEG-6000. The values displayed are the distribution of four replicates. Treatments: -8BOS - Application of Se 8 days before stress (Stressed plants); -4BOS - Application of Se 4 days before stress (Stressed plants); 0OS - Application of Se on the day of stress occurrence (Stressed plants); +4AOS - Application of Se 4 days after stress (Stressed plants); +8AOS - Application of Se 8 days after stress (Stressed plants); +OS-Se - Without Se (Stressed plants); -OS-Se - Without Se (Non-stressed plants).

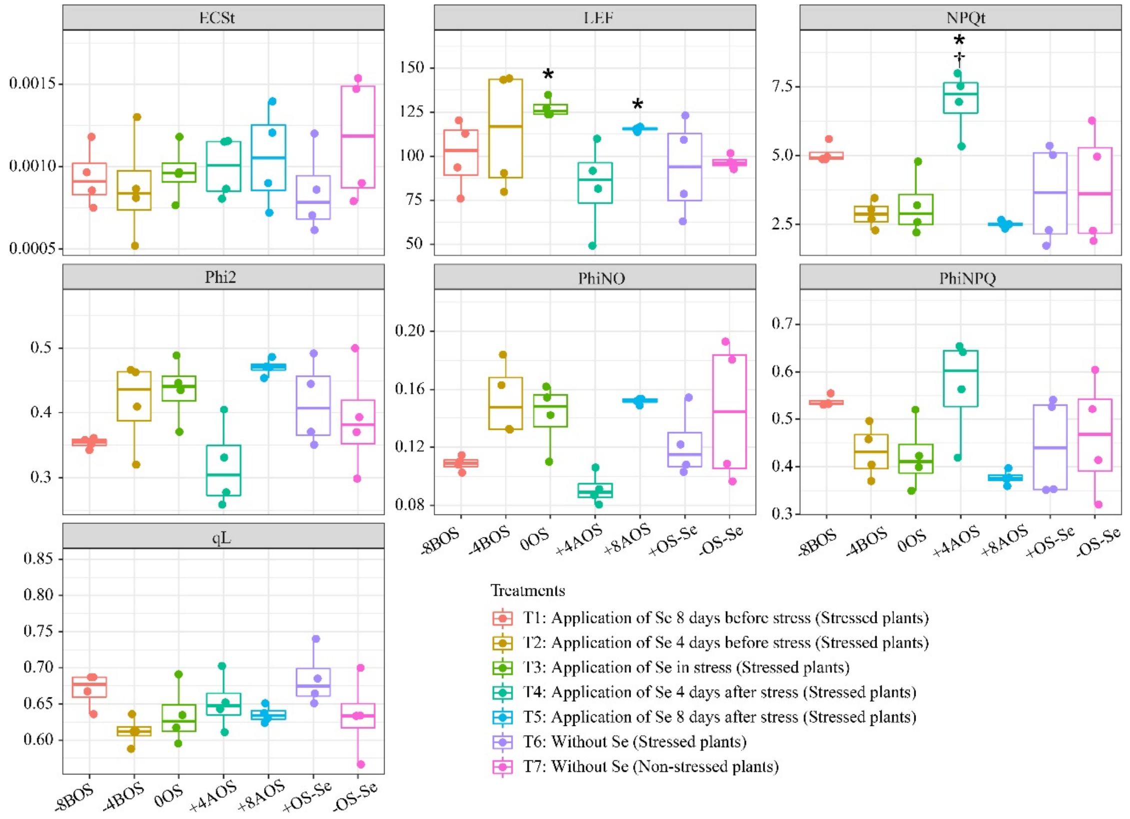

Supplementary Figure S2: Total electrochromic shift (ECSt), total flow of electrons (LEF), total flow of electrons (NPQt), quantum yield of PSII (Phi2), quantum yield of non-regulated energy loss in PSII (PhiNO), quantum yield of regulated non-photochemical energy loss in PSII (PhiNPQ), and fraction of PSII centers which are in the open state (qL) as a result of Se application in *C. arabica* cv. Catuai seedlings under osmotic stress induced by PEG-6000. The values displayed are the distribution of four replicates. Asterisks refer to the significant difference when comparing all treatments with non-stressed plants without Se supply (-OS-Se) ($p < 0.05$). Dagger refers to the significant difference when comparing all treatments with stressed plants without Se supply (+OS-Se) ($p < 0.05$). Treatments: -8BOS - Application of Se 8 days before stress (Stressed plants); -4BOS - Application of Se 4 days before stress (Stressed plants); 0OS - Application of Se on the day of stress occurrence (Stressed plants); +4AOS - Application of Se 4 days after stress (Stressed plants); +8AOS - Application of Se 8 days after stress (Stressed plants); +OS-Se - Without Se (Stressed plants); -OS-Se - Without Se (Non-stressed plants).

This is a provisional file, not the final typeset article

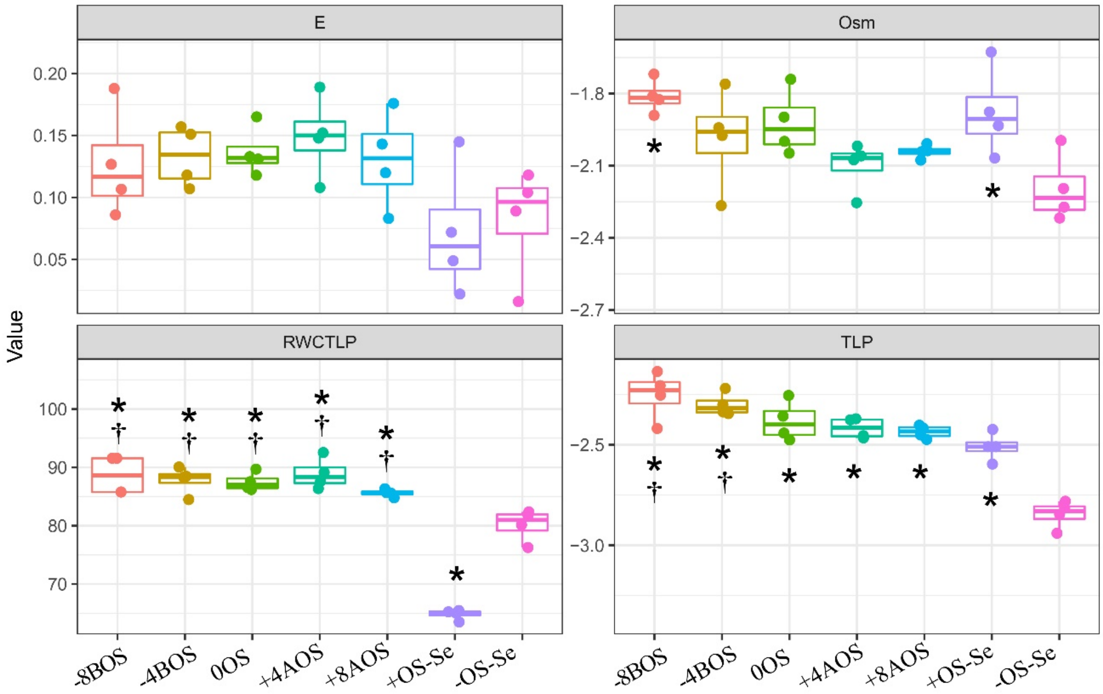

Supplementary Figure S3: Elasticity (E), Osmotic potential (Osm), Relative water content at turgor loss point (RWC_{TLP}), and turgor loss point (TLP) of coffee leaves as a result of Se application in C. arabica cv. Catuai seedlings under osmotic stress induced by PEG-6000. The values displayed are the distribution of four replicates. Treatments: -8BOS - Application of Se 8 days before stress (Stressed plants); -4BOS - Application of Se 4 days before stress (Stressed plants); 0OS - Application of Se on the day of stress occurrence (Stressed plants); +4AOS - Application of Se 4 days after stress (Stressed plants); +8AOS - Application of Se 8 days after stress (Stressed plants); +OS-Se - Without Se (Stressed plants); -OS-Se - Without Se (Non-stressed plants).

Variables	i) Contributions of all variables				Variables	ii) Contributions of reduced number of variables			
	PC1	PC2	PC3	PC4		PC1	PC2	PC3	PC4
PL	7.335	0.739	10.392	7.945	PL	7.064	30.181	4.701	3.123
PH	9.152	0.327	1.796	1.335	PH	-	-	-	-
APX	8.232	5.634	3.123	4.584	APX	17.959	0.142	0.501	4.211
CAT	3.630	2.789	18.581	0.003	CAT	10.394	19.244	0.948	23.059
GR	3.028	7.442	8.588	4.391	GR	12.638	15.202	0.529	0.233
SOD	3.646	14.082	0.767	0.082	SOD	16.491	0.950	7.555	22.579
RS	2.700	0.267	0.016	21.803	RS	-	-	-	-
AA	1.567	0.003	10.418	5.192	AA	-	-	-	-
Pro	11.854	0.405	8.790	0.526	Pro	5.343	20.702	22.985	6.861
TSS	3.719	0.001	9.315	13.493	TSS	-	-	-	-
Starch	9.582	1.860	1.278	2.334	Starch	14.069	0.245	2.407	20.424
Prt	0.432	0.007	3.170	10.096	Prt	0.505	12.252	48.321	13.277
Sac	0.092	0.051	6.206	18.417	Sac	-	-	-	-
Se	11.420	1.475	0.534	0.403	Se	15.536	1.081	12.053	6.231
ECSt	0.132	0.196	0.536	1.079	ECSt	-	-	-	-
LEF	3.474	4.826	3.183	4.346	LEF	-	-	-	-
NPQt	3.443	13.771	4.769	0.419	NPQt	-	-	-	-
Phi2	1.225	15.843	1.842	0.002	Phi2	-	-	-	-
PhiNO	5.915	10.830	3.444	0.195	PhiNO	-	-	-	-
PhiNPQ	1.193	16.279	3.247	0.246	PhiNPQ	-	-	-	-
qL	8.229	3.174	0.003	3.108	qL	-	-	-	-

Supplementary Table S1: Contributions on the first two PC axes of i) all variables and ii) reduced number of variables.

Supplementary Figure S6. Correlation matrix showing Pearson's correlation of physiological, biochemical, and nutritional parameters of Coffea arabica seedlings. The leaf attributes were Leaf Se content (Se); Ascorbate peroxidase (APX); Super-oxide Dismutase (SOD), Catalase (CAT), Glutathione Reductase (GR); Proline; Protein; Li-pid Peroxidation (MDA), and Starch.

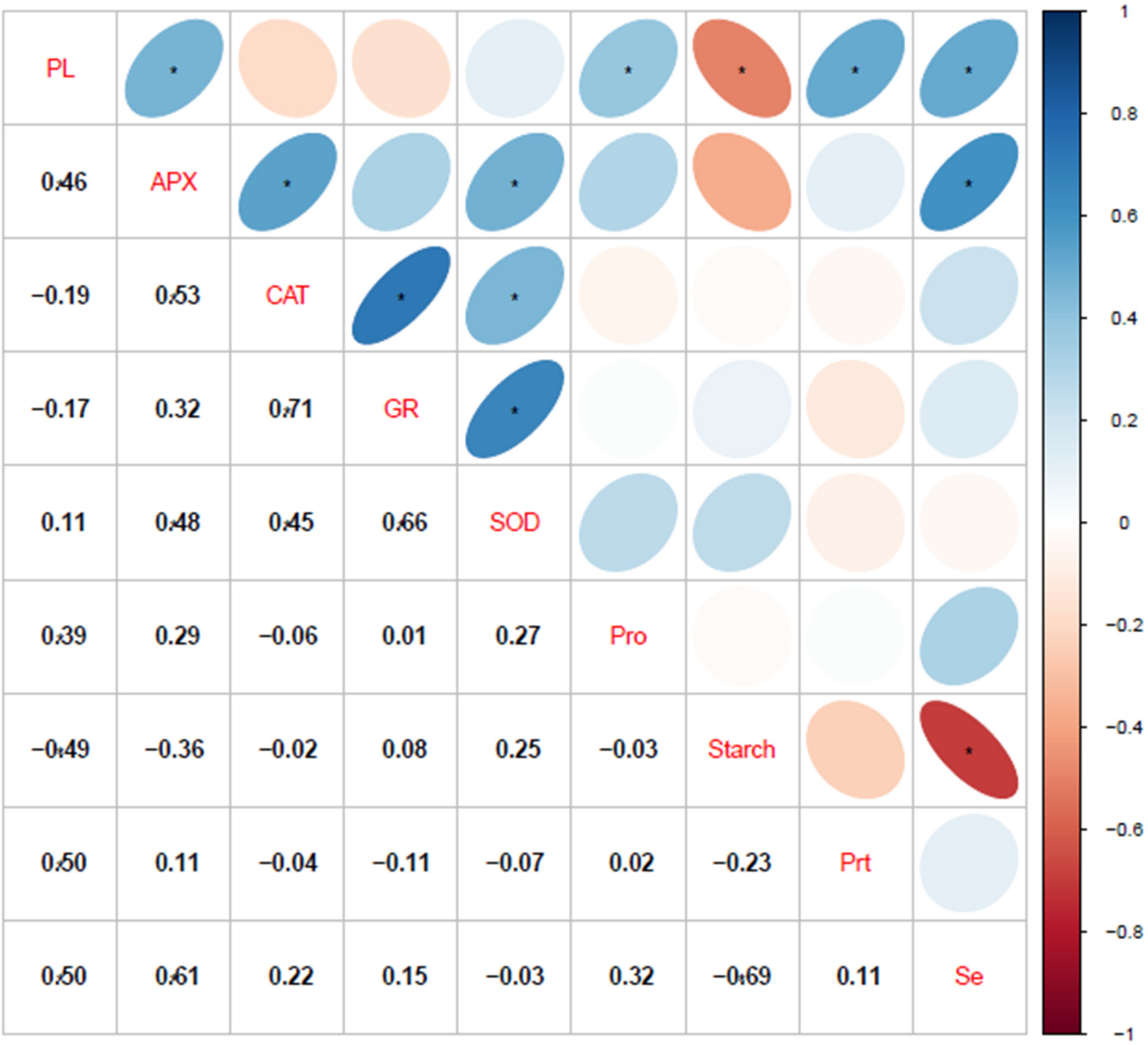

Supplement: Supplementary file 1 [file plants-12-03026-s001.zip › plants-2525923-supplementary.pdf]
